# Datasets: Sensitivity and protein digestion course of proteomic Filter Aided Sample Preparation

**DOI:** 10.1016/j.dib.2019.104530

**Published:** 2019-09-16

**Authors:** Jacek R. Wiśniewski, Katharina Zettl

**Affiliations:** Max Planck Institute of Biochemistry, 82152, Martinsried, Germany

**Keywords:** Proteomic sample preparation, Filter Aided Sample Preparation, Shotgun proteomics, Protein cleavage

## Abstract

Sensitivity of FASP was tested using SDS lysates from HeLa cells and mouse brain. Peptides were analyzed using a QExactive HF-X instrument. Whole cell lysates of Hela cells were processed with FASP using single or double, consecutive or successive, digestion with LysC or trypsin. The generated peptides were analyzed using a LTQ-Orbitrap mass spectrometer. These datasets accompany “Filter Aided Sample Preparation – A Tutorial” (Wiśniewski, 2019).

**Table:** Specifications Table

Subject	Analytical Chemistry
Specific subject area	Sample preparation for proteome analysis
Type of data	Table
How data were acquired	QExactive HF-X or LTQ-Orbitrap mass spectrometer (Thermo-Fisher Scientific, Palo Alto)
Data format	Raw: deposited to the ProteomeXchange Consortium via the PRIDE [2] partner repository with the dataset identifier PXD014288
Parameters for data collection	The mass spectrometers operated in a data dependent mode with survey scans acquired at a resolution of 60,000 at m/z 400. For CID fragmentation (Orbitrap), up to the 10 most abundant precursor ions from the survey scan with charge ≥ +2 within 300-1700 m/z range were selected. For HCD fragmentation (QExactives) up to the top 15 most abundant isotope patterns with charge ≥ +2 from the survey scan (300-1650 m/z) were selected.
Description of data collection	Peptide aliquots were chromatographed on 15 cm (Orbitrap) or 50 cm (QExactives) C_18_-columns. Peptide separation was carried out at 300 nL/min for 60 min (plasma on QExactives) and 95 min (tissues, HeLa and plasma on Orbitrap) using an acetonitrile gradient of 5–30% (v/v) in 0.1% (v/v) formic acid. The columns were thermostated at 60 °C.
Data source location	Max-Planck-Institute of Biochemsitry, 82152 Martinsried, Germany
Data accessibility	Repository name: PRIDE Data identification number: PXD014288 Username: reviewer87998@ebi.ac.uk Password: SxHTiQi6 Direct URL to data:: https://www.ebi.ac.uk/pride/archive/login
Related research article	Author's name: Jacek R Wiśniewski Title: Filter Aided Sample Preparation – A tutorial Journal: Analytica Chimica Acta DOI: https://doi.org/10.1016/j.aca.2019.08.032

**Table:** 

**Value of the Data** • Determination of lower limits of sample amount using FASP • Testing of short times for protein digestion • Data show how consecutive protein digestion increases the yield of conversion of proteins to peptides • The presented data can be used for development of proteomic workflows

## 1 Data

### 1.1 Sensitivity of filter aided sample preparation

**Fig. 1:**
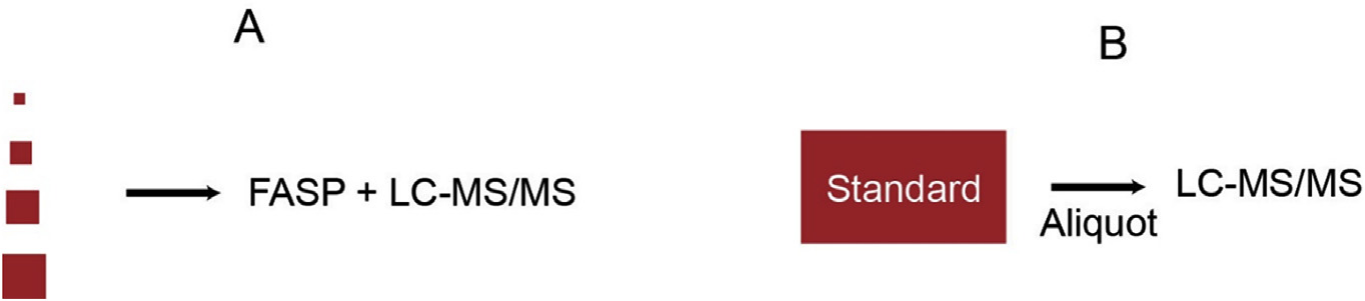
Experimental design of testing FASP sensitivity.

The dataset contain mass spectrometry data obtained through analysis of various cell lysate amounts. Aliquots of mouse brain or HeLa sample containing various amounts of total protein, ranging from 0.25 μg to 10 μg were processed with FASP [3] and analyzed by LC-MS/MS (Fig. 1A) [1]. In parallel a sample containing 100 μg total protein was processed with FASP (Fig. 1B). After quantification of peptides, aliquots containing 0.25 to 10 μg were analyzed by LC-MS/MS. Raw-data were searched using MaxQuant software. The data are shown in Table 1. The complete list of identified peptides and proteins is shown Supplemental Table 1. The raw mass spectrometry data were deposited at PRIDE repository with the dataset identifier PXD014288.

**Table 1:** Identification of peptides and proteins in samples varying in total protein amount.

Sample size (μg)	Brain tissue				HeLa cells			
	FASP		Standard		FASP		Standard	
	peptides	proteins	peptides	proteins	peptides	proteins	peptides	proteins
0.25	4549^a^	1273	4666	1314	6978	1742	3848	1210
0.5	8412	2042	9025	2236	15540	2963	7050	1941
1	12798	2788	19889	3656	21111	3499	22070	3863
2.5	24158	3955	29127	4401	37421	5157	37716	5041
5	29631	4474	33230	4703	41445	5177	43001	5377
10	34026	4760	35610	4853	46770	5626	45516	5513

a Values are averages of two independent experiments. Analysis of peptide mixtures was performed using a QExactive HF mass spectrometer. Complete data are in Supplementary Table 1.

### 1.2 Effect of protein cleavage time on proteomic analysis

**Fig. 2:**
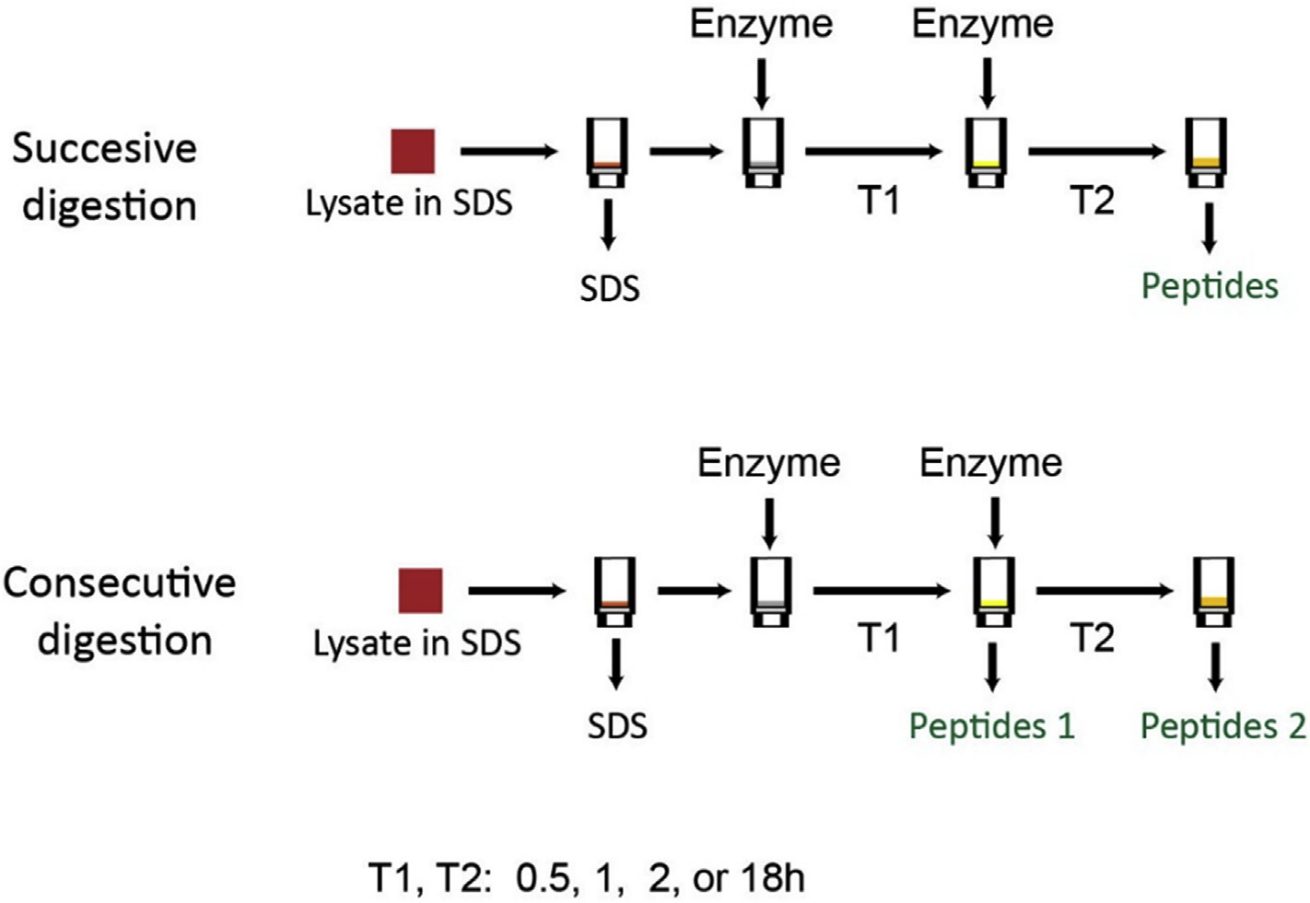
Experimental design of protein cleavage time-course. Always the same enzyme was used for the first and the second digestion.

The dataset contain data that were collected through mass spectrometry analysis of samples cleaved over different times. Aliquots of HeLa lysate containing 50 μg total protein were processed with FASP using either successive or consecutive digestion with endoproteinase LysC or trypsin (Fig. 2). The first and the second digestion were carried out for 0.5, 1, 2 or 18h. The eluted peptides were analyzed by LC-MS/MS [1]. The results are shown in Table 2. The complete list of identified peptides and proteins is shown Supplemental Table 2. The raw mass spectrometry data were deposited at PRIDE repository with the dataset identifier PXD014288.

**Table 2:** Identification of peptides and proteins in samples digested from 0.1, 1, 2 or 18h.

Digestion	Time (h)	Number of peptides and proteins identified				Content of missing cleavage sites	
		LysC		trypsin		LysC %	Trypsin %
		peptides	proteins	peptides	proteins		
First	0.5	6341	1665	8307	1623	8.4	30.3
First	0.5	6210	1620	8151	1586	8.0	30.7
First	1	6481	1670	8656	1687	5.5	25.5
First	1	6321	1680	8396	1652	5.3	25.2
First	2	6520	1690	8648	1724	3.8	19.2
First	2	6510	1699	8691	1785	3.6	21.0
First	18	5910	1621	8137	1764	1.5	9.3
First	18	5716	1586	7974	1765	1.5	8.8
Second consecutive	0.5	4318	1745	6719	2077	2.5	12.7
Second consecutive	0.5	3534	1521	6378	1985	2.6	12.7
Second consecutive	1	3467	1507	5839	1994	2.0	11.2
Second consecutive	1	5716	1624	5822	1983	1.4	11.5
Second consecutive	2	5872	1628	4802	1764	1.6	7.4
Second consecutive	2	4825	1782	4878	1789	2.7	7.0
Second consecutive	18	5073	1788	4034	1286	2.8	4.1
Second consecutive	18	5009	1779	x	x	2.7	x
Doubly succesive	0.5	6769	1699	8400	1711	4.5	20.8
Doubly succesive	0.5	6603	1668	8282	1658	4.5	21.5
Doubly succesive	1	6722	1729	8555	1734	3.3	17.7
Doubly succesive	1	6527	1710	8465	1736	3.1	18.4
Doubly succesive	2	6452	1676	8396	1744	2.4	14.5
Doubly succesive	2	6302	1670	8546	1784	2.4	15.4
Doubly succesive	18	5822	1590	8150	1720	1.6	11.2
Doubly succesive	18	5664	1573	8632	1767	1.4	11.5

Analysis of peptide mixtures was performed using a LTQ-Orbitrap mass spectrometer. Complete data are in Supplementary Table 2.

## 2 Experimental design, materials, and methods

### 2.1 Filter aided sample preparation (FASP)

HeLa cells and thawed pieces (about 50 mg) of mouse brain were homogenized on ice in 2% SDS in 0.1 M Tris-HCl, pH 8.0, containing 0.1 M DTT and lysed as described previously. Sample aliquots containing 50 μg total protein were processed using either FASP or MED FASP method, with some modifications as described in [4].

## 3 Liquid chromatography – tandem mass spectrometry

Analysis of peptide mixtures was performed using a QExactive HF-X or LTQ-Orbitrap mass spectrometer (Thermo-Fisher Scientific, Palo Alto) as described previously in [4], [5], respectively. The raw mass spectrometry files and analysis by MaxQuant software.
